# Unveiling the role of perineural telocytes in mechanosensation, structural insights into their association with herbst and ruffini corpuscles in the quail beak

**DOI:** 10.1038/s41598-025-15900-1

**Published:** 2025-09-12

**Authors:** Soha A. Soliman

**Affiliations:** Department of Histology, Faculty of Veterinary medicine, South Valley University, Qena, Egypt

**Keywords:** Mechanoreceptors, Telocytes, CD34, VEGF, Cell biology, Microscopy

## Abstract

This study investigates the structural organization of telocytes (TCs) in the quail beak, focusing on their association with mechanoreceptors like Herbst and Ruffini corpuscles. By exploring these features, the study aim to expand the understanding of TCs’ role in mechanosensation and sensory modulation. Paraffin sections stained with Hematoxylin and Eosin revealed TCs surrounding both Herbst and Ruffini corpuscles, as well as nerve fibers. Similar findings observed using Mallory Trichrome staining, which highlighted TCs around these sensory structures. Methylene Blue staining further confirmed the presence of TCs in these areas. Semi-thin sections stained with Toluidine Blue also showed TCs encircling the sensory corpuscles, consistent with other techniques. Transmission electron microscopy (TEM) provided detailed ultrastructural insights, revealing TCs near the Herbst corpuscle, with a prominent nucleus, telopodes, and podoms, while TCs around the Ruffini corpuscle exhibited similar features. These findings have clinical relevance, as TCs increasingly recognized for their role in nerve repair and regeneration. Their involvement in sensory modulation suggests potential therapeutic applications for conditions involving nerve injury or sensory dysfunction. Immunohistochemical analysis of quail beak. Using CD34, VEGF, CD21, and CD68 IHC, TCs observed to form a three-dimensional (3D) network around the nerve. The clinical relevance of these findings was significant, as TCs increasingly recognized for their role in nerve repair and regeneration. Their involvement in sensory modulation suggests potential therapeutic avenues for conditions related to nerve injury or sensory dysfunction. Ongoing research into TCs will further deepen our understanding of their functions in sensory systems and may pave the way for novel treatments for sensory disorders.

**Supplementary Information:**

The online version contains supplementary material available at 10.1038/s41598-025-15900-1.

## Introduction

Telocytes (TCs), a unique type of interstitial cells, have emerged as a subject of significant interest in cellular and molecular biology due to their distinctive morphological features and versatile functional roles in various tissues and organs. TCs characterized by their small cell bodies and extremely long, thin, branching extensions called telopodes, which form intricate networks in the extracellular matrix^1,2^. Telocytes exhibit distinct moniliform structures, which are composed of specialized podoms^3–5^. These cells identified in a wide range of tissues, including the dermis^6^skeletal muscle^4,7,8^gastrointestinal tract^9,10^and reproductive system^11,12^. Recent studies have highlighted the critical roles of telocytes in intercellular signaling and their ability to interact with neighboring cells through direct contact^13^ or via extracellular vesicles underscores their importance in cellular communication^14^ and tissue coordination^15,16^.

The quail (*Coturnix coturnix*) has become a widely used model organism in biological and biomedical research due to its unique advantages, including its small size, rapid life cycle, high reproductive rate, and physiological similarities to other vertebrates, including humans. Quail reach sexual maturity in about 6–8 weeks and produce a large number of eggs year-round, making them ideal for studies requiring large sample sizes and multiple generations in a short time frame^17^. Their embryos are easily accessible for experimental manipulation, such as gene editing, tissue grafting, and live imaging, which has established quail as a popular model for studying embryogenesis, tissue regeneration, and developmental processes^18,19^. Additionally, quail share many physiological and genetic traits with mammals, making them valuable for studying human diseases, cardiovascular development, neurogenesis, and organogenesis. The quail beak, with its well-defined mechanoreceptors^20^. In neuroscience, mechanoreceptors serves as an excellent model for investigating sensory processing^21^and nerve regeneration^22,23^. Advances in genetic engineering have further enabled the creation of transgenic quail, allowing researchers to study gene function and regulation in vivo, as well as model human diseases^17^. Moreover, quail are cost-effective and easy to maintain compared to larger animal models, making them a practical choice for diverse research applications. Their versatility spans fields such as developmental biology, regenerative medicine, genetics, and neuroscience, with recent studies continuing to highlight their potential for addressing fundamental biological questions and developing translational applications^18,24^.

In this study, we examine the morphology of TCs in the quail beak, a model system well-suited for exploring sensory structures. Using a combination of histological techniques, including Hematoxylin and Eosin, Mallory Trichrome, Methylene Blue, and Toluidine Blue staining, we visualize TCs in relation to mechanoreceptors and nerve fibers. Additionally, Transmission Electron Microscopy (TEM) provides high-resolution insights into the ultrastructural features of TCs. The results consistently demonstrate the presence of TCs surrounding sensory receptors, as well as nerve fibers, highlighting their possible role in relation to sensory structures.

This study aims to provide a comprehensive understanding of the structural organization of TCs in the quail beak around the mechanoreceptors. By elucidating these features, we contribute to the broader knowledge of TCs function in mechanosensation and offer potential insights into peripheral nerve function and sensory modulation. The findings from this study may also have implications for understanding sensory disorders and developing therapeutic strategies for peripheral nerve injuries.

## Materials and methods

All animal experiments follow the ARRIVE guidelines and carried out in accordance National Research Council (US)^25^.

### Ethical approval

The methodology used in this study approved by the National Ethics Committee of South Valley University and the veterinary authorities in Qena Province, Egypt. All procedures carried out in compliance with applicable policies and guidelines.

### Sampling

The research conducted using apparently healthy quail birds *(Coturnix coturnix japonicum).* Beak samples collected from birds aged 1 week, 2 weeks, and 6 weeks. This study used isoflurane anesthesia (5% induction, 2–3% maintenance in 100% O₂) administered via a precision vaporizer in an induction chamber. Anesthesia depth was confirmed by loss of righting reflex and absent response to toe pinch^26^. For euthanasia, quail were rapidly decapitated at the atlanto-occipital junction. The protocol was approved by the Institutional Animal Care Committee. The upper beak carefully dissected and rinsed with saline before being fixed.

For light microscopy, the embryos promptly fixed in 10% neutral buffered formalin and then immersed in Bouin’s solution for 30 min. The fixed samples underwent dehydration through a graded series of ethanol concentrations (70%, 80%, 90%, and 100%). Following dehydration, the samples cleared using methyl benzoate and embedded in Paraplast (MilliporeSigma, St. Louis, MO, USA) prior to impregnation. The processing times for paraffin embedding detailed in Table 1.

**Table 1:** The processing time of the samples in paraffin embedding techniques.

Age process	5d	8d	15d
1-Fixation			
A-NBF	8 h	13 h	**24 h**
B_Bouin’s solution	1/2 h	1/2 h	**1/2 h**
2-dehydration Alcohol70%I	2 h	2 h	**2 h**
Alcohol 70%II	2 d	2d	**2d**
Alchol70%III Alchol80%	1 h	2 h	**2 h**
Alchol90%	1 h	2 h	**1 h**
Alchol100%	1/2 h	1/2 h	**1/2h**
Alchol100%	1/2 h	1/2 h	**1/2h**
3-clearing with methylebenzot			
MBI	1 h	1 h	**1 h**
MB II	12 h	12 h	**12 h**
MBIII	12 h	12 h	**12 h**
4-embedding in paraffin:			
P I	2 h	2 h	**1/2h**
P II	2 h	2 h	**1/2h**
P III	**4 h**	**4 h**	**1 h**

*NBF* (neutral buffer formalin), *h*, hours; *d*, days; *MB I*, methyl bonzoate1, *MB II*, methyl benzoate II; *PI*, paraffin I; *P II*, paraffin II; *P III*, paraffin III.

### Histological examination

Paraffin sections with a thickness of 5 μm obtained using a Leica microtome (model number: RM2125, manufactured by Leica Microsystems, Wetzlar, Germany). The sections stored in an incubator at 40 °C to ensure they completely dry. Hematoxylin and eosin used to stain the sections for a general histological examination^27^. The sections also stained by Mallory trichrome, Methylene blue, Mallory triple, Crossman’s trichrome, Giemsa, and Weigert van Gieson stain^27^.

### Preparations of resin embedding samples for semi-thin sections

Karnovsky’s fixative utilized for resin-embedding samples. The fixative prepared by combining 50 mL of phosphate buffer, 50% glutaraldehyde, 25% paraformaldehyde, and 30% distilled water, with 10 mL of each component. Samples trimmed into 2.0 to 3.0 mm pieces. These samples then fixed and stored at 4 °C overnight (Table 2)**.**

**Table 2:** Components of the fixative.

Fixative	Components	Amount
Karnovsky fixative	Paraformaldehyde, 25% freshly prepared	10 ml
	Glutaraldehyde 50%	10 ml
	Na-Phosphate buffer (0.1 M, pH 7.4)	50 ml
	Distilled water	30 ml
N a-Phosphate buffer (0.1 M, pH 7.4)	Solution A	
	Na2HPO4 2H2O	17.02 gm
	Distilled water	600 ml
	Solution B	
	NaH2PO4 H2	6 gm
	Distilled water	200 ml
	Using solution	
	Solution A	580 ml
	Solution B	219 ml
Citrate-buffer (pH 6.0)	Solution A	
	Citrate C6H8O7 H2O	21 g
	Distilled water	1 L
	Solution B	
	Sodium citrate Na3C6H5O7 2H2O	29.41 g
	Distilled water	1 L
	Using solution	
	Solution A	9 ml
	Solution B	41 ml
	Distilled water	Add 500 ml

The preparation process consisted of multiple steps^28^: the samples postfixed using osmium tetroxide, embedded in resin, cleaned, and polymerized in an oven at 60 °C. For resin impregnation, a combination of pure resin and alcohol employed, with propylene oxide assisting in the embedding process. Initially, a 1:1 ratio of epoxy resin and propylene oxide applied for approximately 30 min, followed by a three-hour treatment with pure epoxy resin. The epoxy resin mixture prepared by mixing 5 mL of Araldite, 5 mL of EMbed 812, and 12 mL of dodecenylsuccinic anhydride (DSAA). After embedding, the resin mixture heated at 60 °C to polymerize the samples, with the addition of an accelerator (2,4,6-Tris[dimethylaminomethyl] phenol; 1.5%). The blocks then incubated for three days at different temperatures: one day at 60 °C, one day at 70 °C, and one day at 75 °C^27^.

Semithin sections, measuring 1 μm in thickness, prepared using the Ultracut E ultramicrotome manufactured by Reichert-Leica in Germany. These sections subsequently stained with toluidine blue^1,10^and examined using a Leitz Dialux 20 microscope and a Canon PowerShot A95 digital camera.

Ultra-thin sections were prepared and stained with uranyl acetate and lead citrate to enhance contrast for detailed analysis. These stained sections, mounted on grids, were examined using a JEOL100CX II transmission electron microscope at South Valley University’s central laboratory in Egypt. This process enabled the visualization and analysis of ultrastructural details at a nanometer scale.

## Immunohistochemistry staining

### Immunohistochemistry staining procedures for CD34, and CD68

The immunohistochemistry staining for CD34 and CD68 carried out using the Lab Vision Ultra Vision Detection System in combination with anti-polyvalent, horseradish peroxidase/3,3´-diaminobenzidine (DAB), Ready-to-Use reagent (Thermo Fisher Scientific, Waltham, MA, USA). The antigen localization achieved through the avidin-biotin complex technique, following the manufacturer’s protocol^29^.

The immunohistochemical protocol followed is outlined below, as referenced by several studies^30–32^: Initially, 5 μm thick paraffin sections rinsed three times with a phosphate-buffered saline (PBS) solution at pH 7.4 for five minutes per wash. Subsequently, the sections dewaxed using xylene and rehydrated through graded ethanol solutions. To block endogenous peroxidase activity, the slides incubated in hydrogen peroxide at room temperature. After a 10-minute wash under running tap water, antigen retrieval performed by placing the slides in a 10 mmol sodium citrate buffer (pH 6.0) at 95–98 °C for 20 min. Following the heat treatment, the slides cooled to room temperature for 20 min and washed with PBS (pH 7.4) three times for five minutes each. To prevent nonspecific background staining, blocking performed using Ultra V Block (Thermo Fisher Scientific) for five minutes at room temperature, limiting the staining duration to 10 min.

After primary antibody application (as specified in Table 3), the sections incubated overnight at 4 °C. The sections then washed with PBS (pH 7.4) three times for five minutes each. Subsequently, the secondary antibody (Table 3) applied and allowed to incubate at room temperature for 10 min. After another round of washing, the slides treated with streptavidin-peroxidase complex (Thermo Fisher Scientific UK and Lab Vision Corporation) at room temperature for 10 min. To visualize the bound antibodies, DAB plus substrate and DAB plus chromogen mixed and applied to the slides for five minutes. The staining process occurred in a humid chamber to prevent drying. The counterstaining done with Harris hematoxylin for 30 s. Finally, the sections dehydrated twice in 100% ethanol for five minutes each, cleared in xylene, and mounted with DPX (dibutylphthalate polystyrene xylene) mounting solution. The immunohistochemical staining then analyzed using a Leitz Dialux 20 microscope and a Canon PowerShot A95 digital camera.

**Table 3:** Identity, sources, and working Dilution of antibodies used in immunohistochemical studies. Antibodies used that showed reactivity in avian species.

	Supplier	Origin	dilution	Incubation	Antigen retrieval	Biotinylated secondary antibody supplier
CD34	Mouse anti chicken CD34 (Bio rad)	Mouse anti chicken CD34 monoclonal antibody (Clone: AV138) (Cat.no MBS224490)	1:100	Over night	Boiling in citrate buffer (pH 6.0), 20 min	Goat anti-mouse IgG (H + L) secondary antibody Catalog # 31,569 Dilution; 1:100 One hour at room temperature
CD68 (Macrophage marker) Ab-3 (Clone KP1)	Mouse anti-CD68 thermo fisher scientific lab vision Corporation, Fremont, USA	Mouse monoclonal antibody Cat. #MS-397-R7	1:100	Over night	Boiling in citrate buffer (pH 6.0), 20 min	
VEGF	Rabbit anti -VEGF (Invitrogen by Thermo Fisher Scientific Waltham, MA, USA)	Rabbit VEGF Polyclonal Antibody (clone: RB-222- P0) (Cat.no PA1- 21796)	1:100	Over night	Boiling in citrate buffer (pH 6.0), 20 min	Goat anti-rabbit secondary antibody (cat. no. K4003, EN Vision + TM System Horseradish Peroxidase Labelled Polymer; Dako) ready to use 30 min at room temperature
CD21	Rabbit anti- CD21 antibody (Abcam)	Rabbit Anti-CD21 antibody monoclonal SP186](ab227662)	1:100	Over night	Boiling in citrate buffer (pH 6.0), 20 min	

### Immunohistochemical procedures for vascular endothelial growth factor (VEGF) and CD21

The two-step immunohistochemistry staining for VEGF and CD21 performed using the Dako EN Vision + Single Reagent (HRP, Mouse) (Agilent Technologies, Inc., Santa Clara, CA, USA), as recommended by^33^. The process began with dewaxing and rehydrating paraffin-embedded Sect. (5 μm thick) and washing them three times with PBS (pH 7.4) for five minutes each. To block endogenous peroxidase activity, the sections incubated in a 3% hydrogen peroxide solution in methanol at room temperature for 20 min, followed by washing under running tap water for 10 min. Antigen retrieval achieved by placing the slides in a 10 mmol sodium citrate buffer (pH 6.0) and heating them in a tap water bath at 95–98 °C for 20 min. After cooling to room temperature for 20 min, the slides washed three times with PBS (pH 7.4). To prevent nonspecific background staining,

The primary antibody (as referenced in recent studies on avian species, Chen et al., 2014) then applied to the sections. The antibodies used in the immunohistochemical process listed in Table 3. After incubating the slides with the primary antibody, the secondary antibody applied for 30 min at room temperature. The sections washed again with PBS (pH 7.4) and treated with DAB substrate-chromogen for 5–10 min at room temperature. Following the incubation, the slides rinsed three times with PBS, and a brown precipitate formed at the antigen sites.

To counterstain the slides, Harris hematoxylin applied for 30 s. The sections then dehydrated twice in 90% and 100% ethanol for five minutes each, cleared with xylene, and mounted with DPX. The stained sections examined under the Leitz Dialux 20 microscope and captured with the Canon PowerShot A95 digital camera. Negative control samples prepared by omitting the primary antibody from the staining procedure to confirm the specificity of the staining.

### Statistical analysis

The current study used serial sections taken form 5 birds. Statistical analyses were performed using GraphPad Prism software (version 8.0.1, GraphPad Software Inc., San Diego, CA). Intergroup comparisons between control and saline-treated samples were assessed by one-way ANOVA, with statistical significance set at *p* < 0.05. Post hoc pairwise comparisons of receptor expression means were conducted using Tukey’s multiple comparisons test.

## Results

**Fig. 1:**
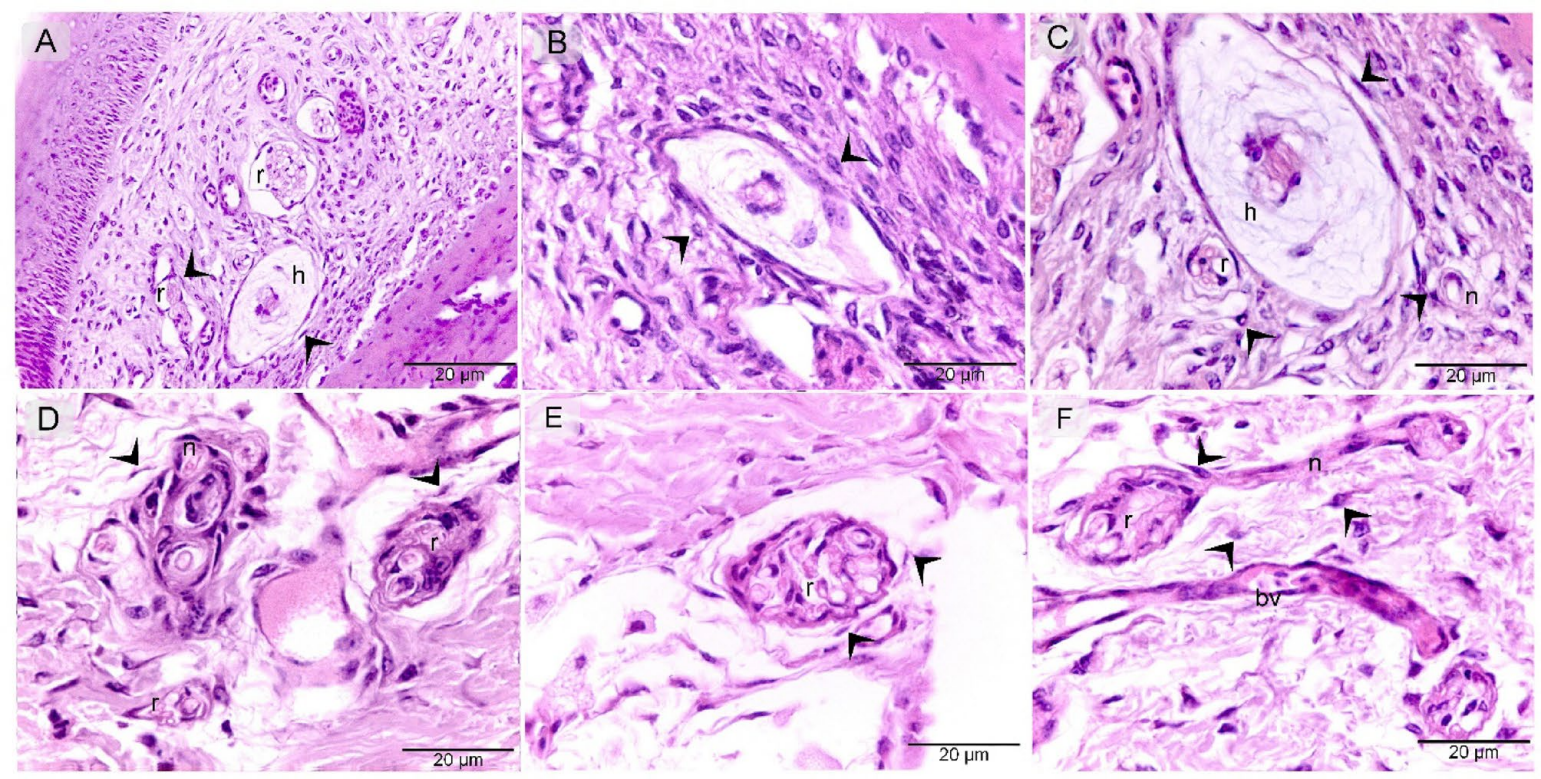
Recognition of TCs H&E. Paraffin section of quail beak stained with H&E. TCs (arrowheads) located around Herbest (h), Ruffni (r) corpuscles, nerve fibers (n). Note. Blood vessels (bv).

**Fig. 2:**
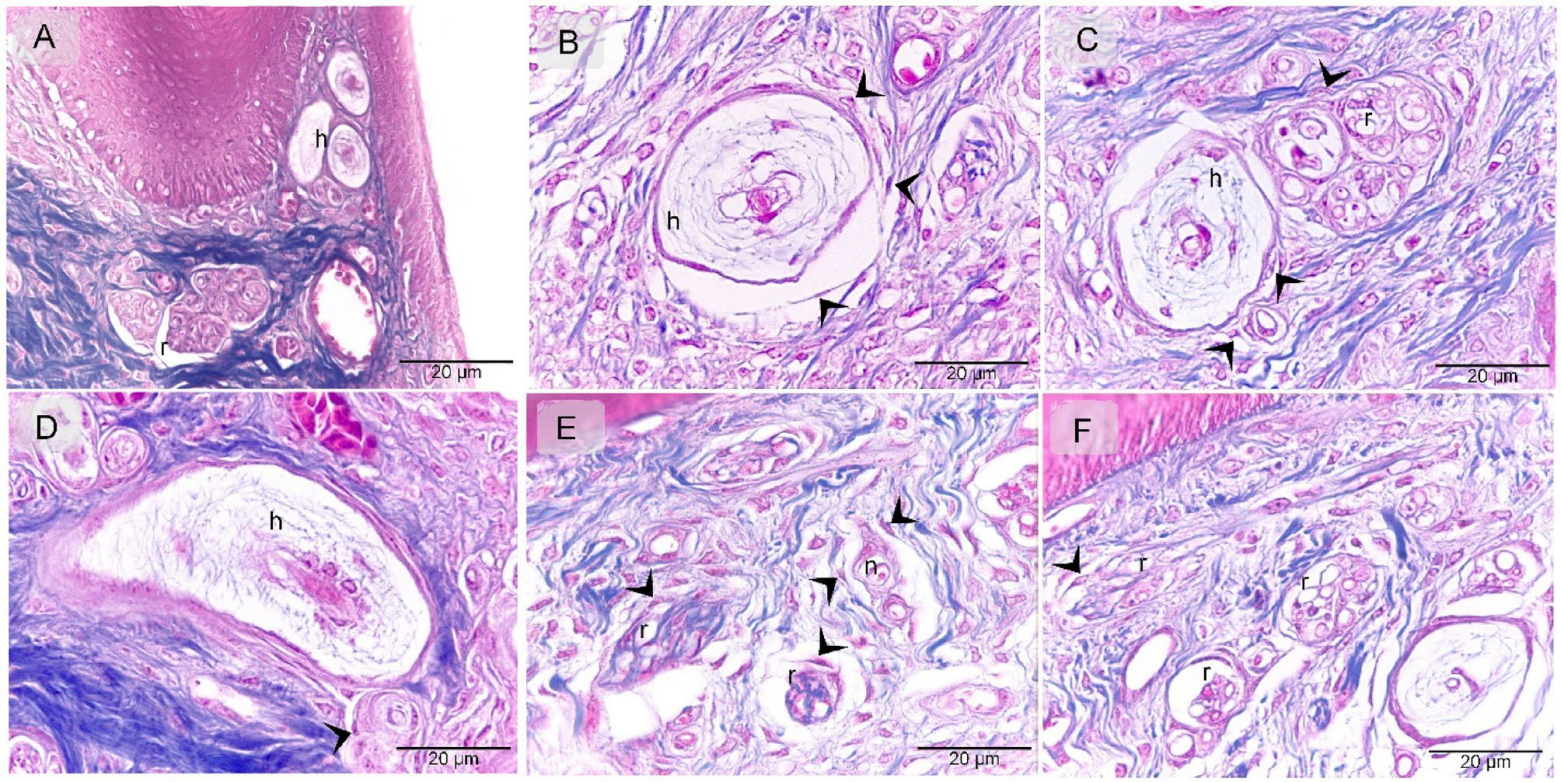
Recognition of TCs Trichrome. Paraffin section of quail beak stained with trichrome. TCs (arrowheads) located around Herbest (h), Ruffni (r) corpuscles, nerve fibers (n).

**Fig. 3:**
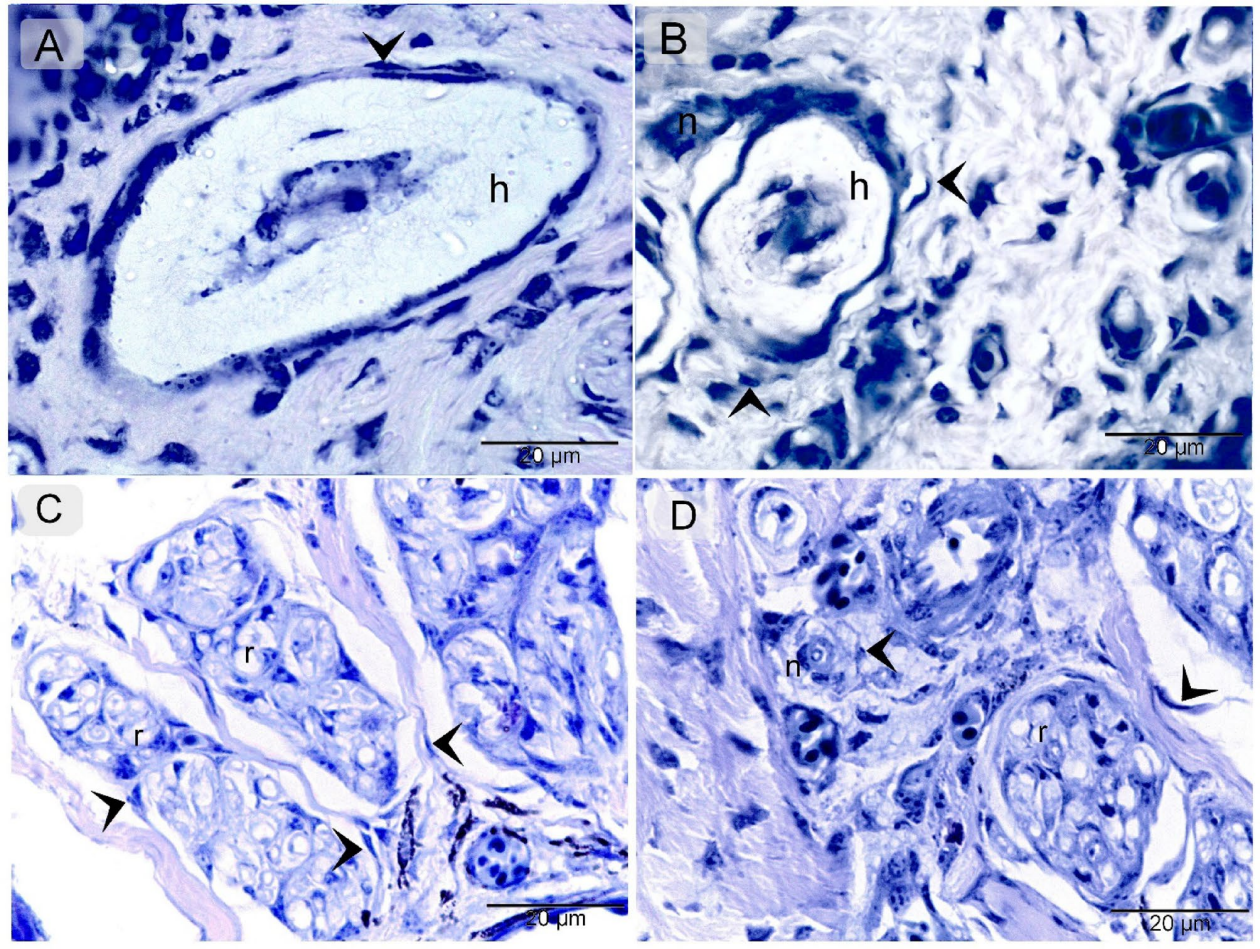
Identification of TCs methylene blue. Paraffin section of quail beak stained with methylene blue. TCs (arrowheads) located around Herbest (h), Ruffni (r) corpuscles, nerve fibers (n).

The results obtained through various staining techniques demonstrate the distribution pattern of telocytes (TCs) along the entire course of the nerve fibers, including nerve ending, while also revealing characteristic morphological features of TCs in the cranial third of the quail beak. Paraffin sections of the quail beak stained with Hematoxylin and Eosin, with TCs located around the Herbest and Ruffini corpuscles, as well as nerve fibers (Fig. 1A-F). Mallory Trichrome demonstrate similar findings to Fig. 1 with TCs around the Herbest and Ruffini corpuscles, as well as nerve fibers (Fig. 2A-F). Methylene Blue, further confirming the presence of TCs surrounding the Herbest and Ruffini corpuscles, along with nerve fibers (Fig. 3A-D).

**Fig. 4:**
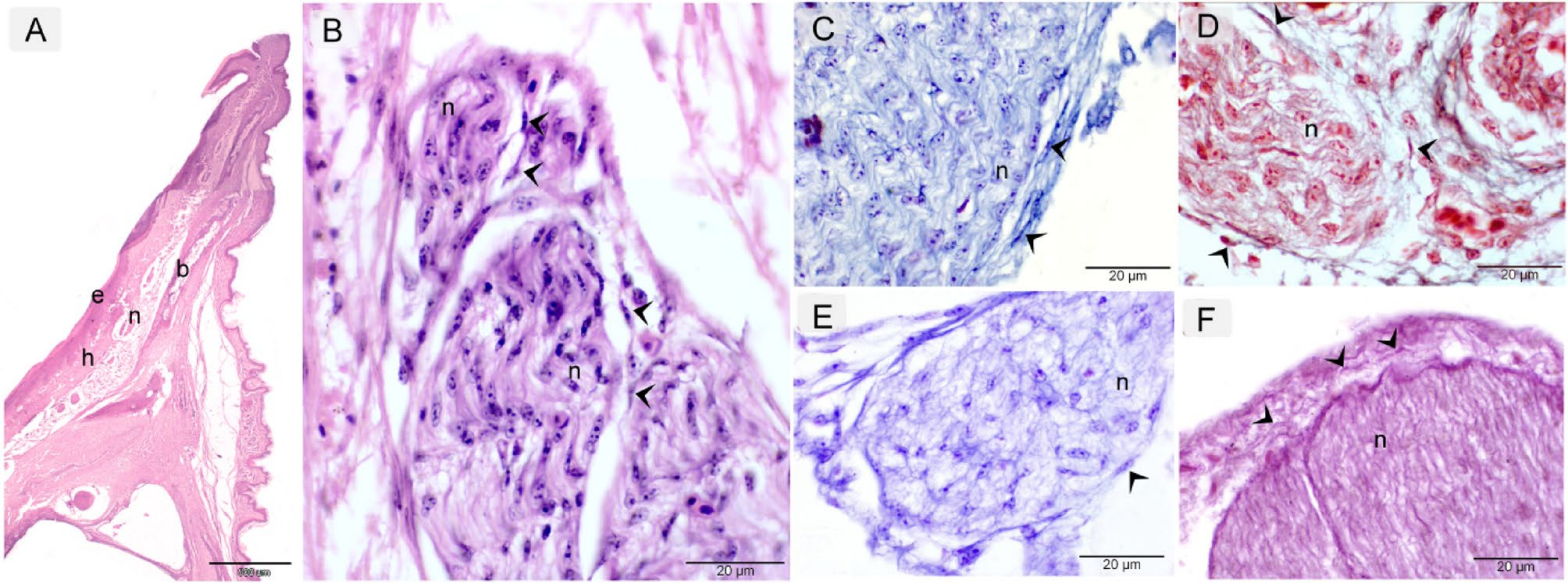
Histochemical properties of TCs ophthalmic branch of trigeminal nerve (cranial nerve). Paraffin sections of the quail beak stained with H&E (**A**, **B**), Mallory triple (**C**), Crossman’s trichrome (**D**), Giemsa (**E**), and Weigert van Gieson stain (**F**). Telocytes (TCs) (arrowheads) observed surrounding a nerve trunk (n). The quail beak structure consists of an outer epithelium (e), underlying bone (b), and the lamina propria, which contains nerves (n).

The quail beak exhibits a stratified architecture consisting of three principal layers: (1) a keratinized epithelial surface (Fig. 4A), (2) an underlying osseous core, and (3) an intervening lamina propria that harbors mechanosensory Herbst corpuscles and extensive neural networks (Fig. 4B). The histochemical analysis revealed telocytes (TCs) in close association with the ophthalmic branch of the trigeminal nerve, demonstrated through a panel of specialized stains: Hematoxylin and Eosin (H&E) Stain (Fig. 4A, B), Mallory Triple Stain (Fig. 4C), Crossman’s Trichrome Stain (Fig. 4D), Giemsa Stain (Fig. 4E), and Weigert Van Gieson Stain (Fig. 4F).

**Fig. 5:**
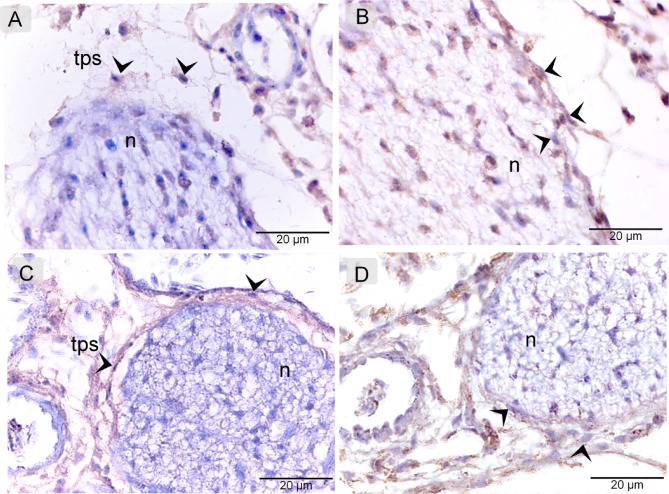
Immunohistochemical Properties of Perineural TCS. Paraffin sections of quail beak were immunostained for CD34 (**A**), VEGF (**B**), CD21 (**C**), and CD68 (**D**). TCs (arrowheads) formed a 3D network around the nerve (N). note Tps (telopodes).

TCs identification confirmed using immunohistochemically stained paraffin sections of the quail beak. They expressed CD34 (Fig. 5A) and VEGF (Fig. 5B). They also showed strong immunoreactivity for CD21 (Fig. 5C) and CD68 (Fig. 5D). TCs observed to form a three-dimensional (3D) network surrounding the nerve.

**Fig. 6:**
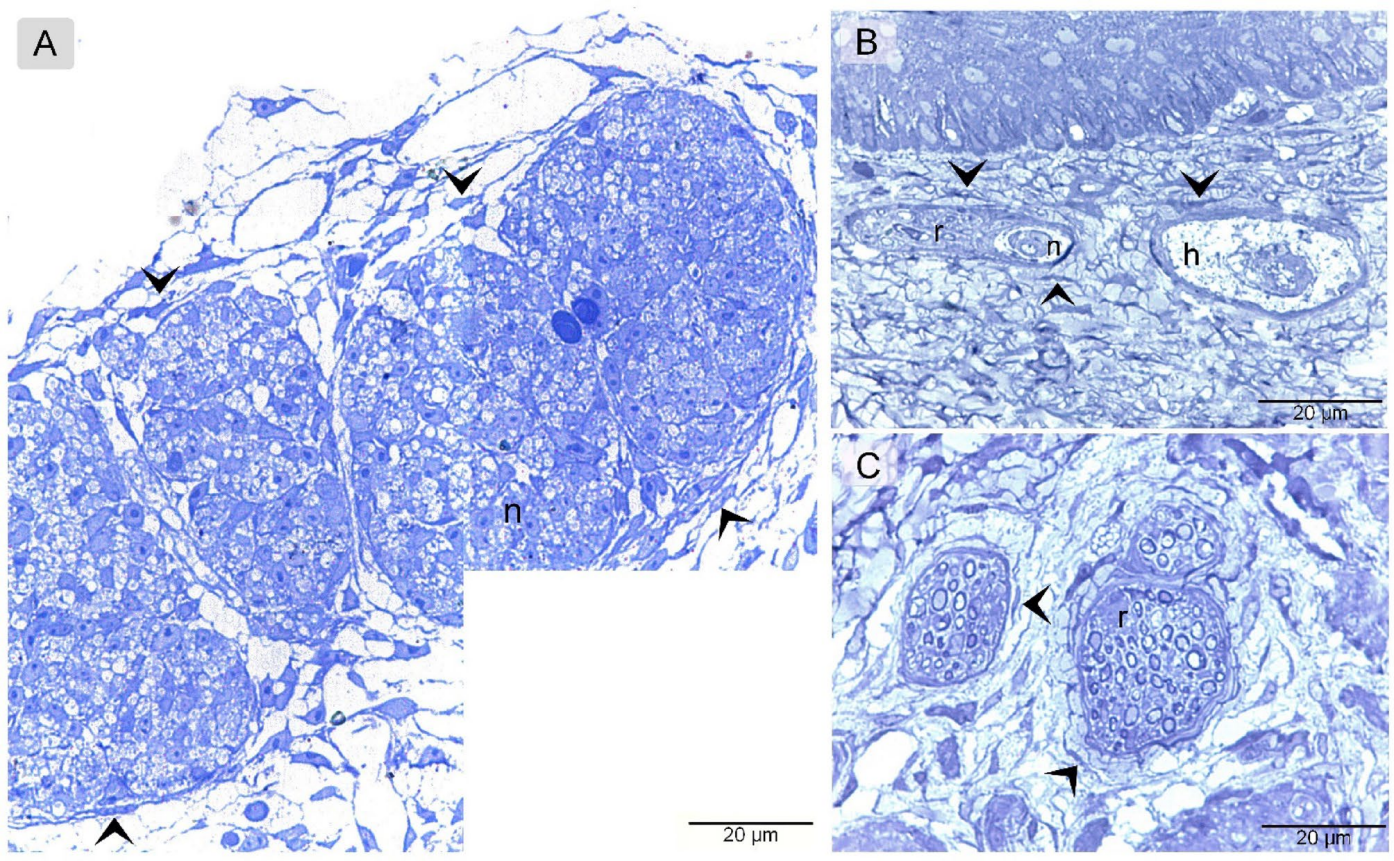
Identification of TCs toluidine blue. Semi-thin section of quail beak stained with toluidine blue. TCs (arrowheads) located around nerve (n), Herbest (h), and Ruffni (r) corpuscles.

Semi-thin sections of the quail beak stained with Toluidine Blue reveals TCs surrounding the Herbest, Ruffini corpuscles and nerve fiber, similar to the other staining techniques (Fig. 6A-C).

**Fig. 7:**
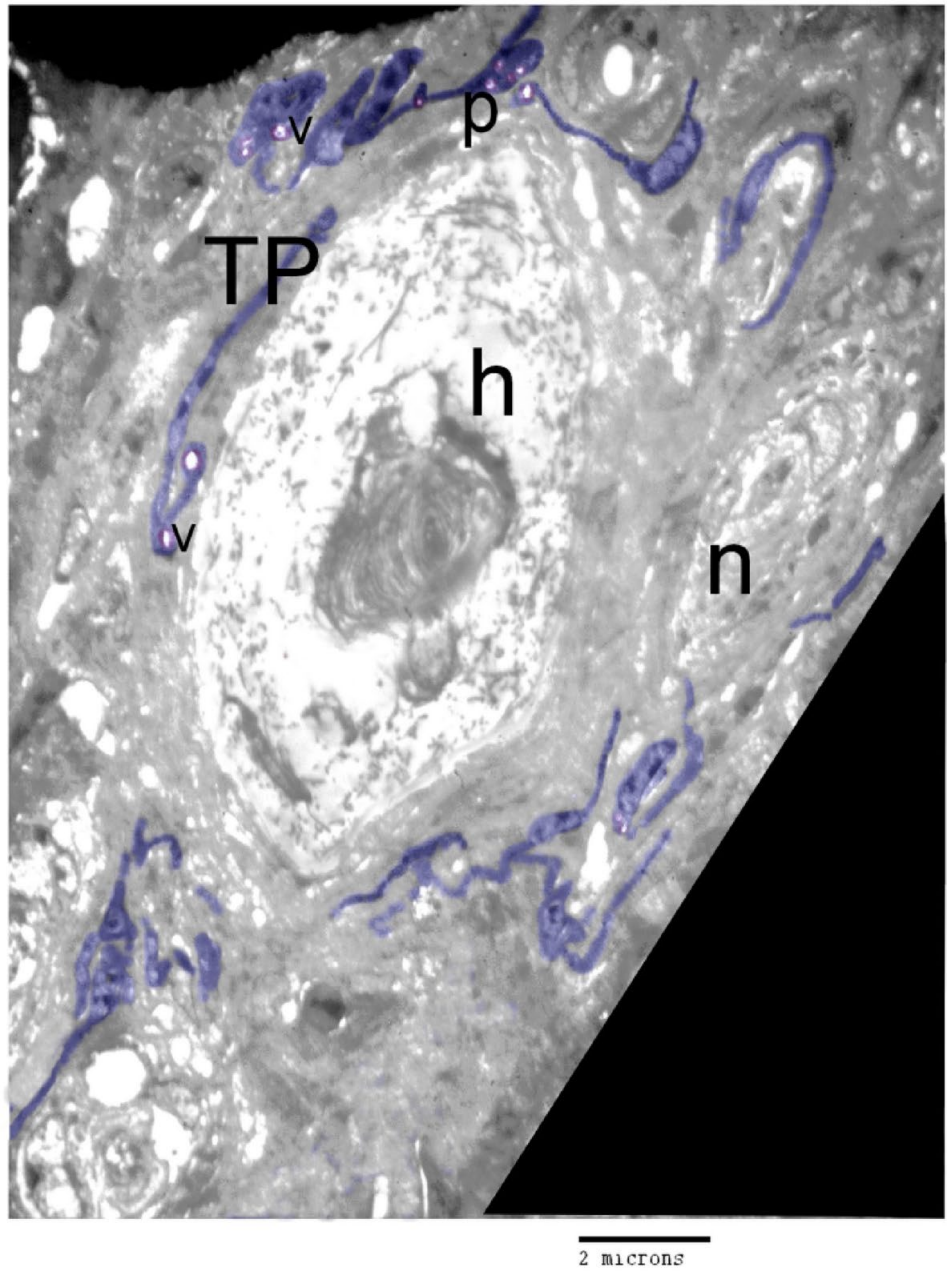
Organization of perineural TCs using TEM. TCs located around the Herbest corpuscle. They formed of cell body with prominent nucleus and telopodes (TPs), podoms (p), secretory vesicle (v) and nerve (n).

**Fig. 8:**
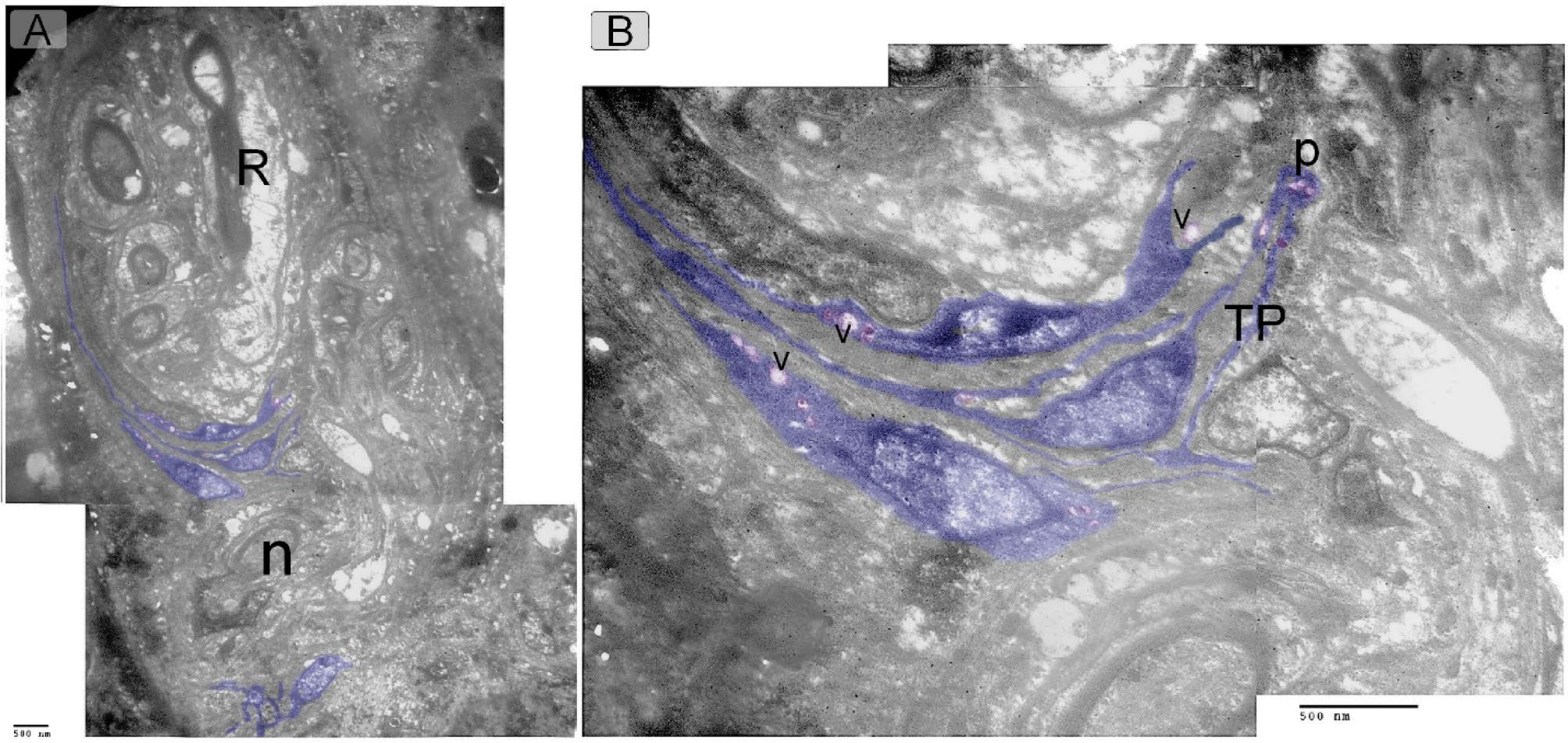
Ultrastructure features of perineural TCs. TCs located around the Ruffini corpuscle (R). They formed of cell body with prominent nucleus and telopodes (TPs), podoms (p), secretory vesicle (v) and nerve (n).

TEM provides a detailed view of TCs located around the Herbest corpuscle, showing their cell body with a prominent nucleus, as well as telopodes and podoms forming the structure of the TCs (Fig. 7). TCs surrounding the Ruffini corpuscle, again displaying the characteristic cell body with a prominent nucleus and the formation of telopodes and podoms (Fig. 8A, B).

Quantitative analysis revealed distinct telocyte densities associated with different mechanosensory structures (Table 4).

**Table 4:** Telocyte densities associated with mechanosensory units.

Structure	Telocyte density (Approx.)
Nerve bundles	15–22 telocytes/20 mm²
Herbst corpuscle	11–17 telocytes/20 mm²
Ruffini corpuscle	14–20 telocytes/20 mm²

## Discussion

The results observed through various staining techniques have provided valuable insights into the spatial distribution and morphology of TCs in the quail beak, particularly in their association with the Herbest and Ruffini corpuscles. TCs, located around these sensory structures, play a pivotal role in supporting and modulating the activity of nerve fibers, contributing to the overall sensory function of mechanoreception.

A critical feature of TCs is their unique morphology, particularly the formation of telopodes and podoms, which are extensions of the cell body. These structures are essential for the interaction of TCs with surrounding nerve fibers and sensory structures. Telopodes are long, thin processes that extend from the cell body. Telopodes form branching 3D system that makes in close proximity to with the nerve endings. These morphological adaptations enable TCs to establish close physical and functional associations with nerve fibers and mechanoreceptors, facilitating their role in sensory processing.

The primary role of TCs is to provide molecular microenvironments and structural support to the nerve fibers they encase. TCs locate in association with unmyelinated nerve endings, particularly those found in sensory organs. This association is critical for sensory function, where TCs help maintain the integrity of nerve fibers, support their growth and regeneration^34^ to ensure the transmission of sensory signals. Moreover, TCs express nerve growth factor^35^ which activates Schwann cells^36^. Studies by Salzer^37^ have outlined the multifaceted role of Schwann cells in peripheral nerve function, indicating that these cells play a vital role not only in myelination but also in modulating sensory input and contributing to nerve regeneration.

Telocytes are known to secrete exosomes as part of their paracrine signaling mechanism. These exosomes are formed through the endosomal sorting complex required for transport (ESCRT) pathway and are released into the extracellular space upon fusion of multivesicular bodies (MVBs) with the plasma membrane^38^. Telocyte-derived exosomes contain a diverse cargo, including microRNAs (miRNAs), mRNAs, proteins, and growth factors, which can modulate the behavior of neighboring cells^39^.

TCs found in close association with nerves, sensory nerve endings, ganglia and the intestinal autonomic nervous system^40^sensory nerve endings^41^. The current study reveals the existence of TCs in association with sensory nerve endings, including mechanoreceptors such as the Herbest and Ruffini corpuscles. These mechanoreceptors are key sensory structures that detect mechanical stimuli, such as pressure, stretch, and vibration, essential for tactile sensitivity and proprioception^42^. For mechanoreceptors like the Herbest and Ruffini corpuscles detect pressure, vibrations, fine textures and stretch^43,44^. The close relationship between TCs and mechanoreceptors like the Herbest and Ruffini corpuscles suggests that TCs may play an active role in modulating sensory function. In particular, TCs may be involved in regulating mechanoreceptive responses, influencing the activity of sensory receptors based on the context of the stimuli. This modulation could involve changes in the extracellular environment, such as the release of signaling molecules or neurotrophic factors, which affect the activity of surrounding nerve fibers^45^. The presence of TCs around sensory endings may be crucial for their sensitivity to mechanical stimuli, aiding in the adaptation of the sensory system to changes in the mechanical environment. These findings underscore the dynamic, supportive role of TCs in ensuring proper mechanosensory function and response to environmental changes. The current study suggests interaction between TCs and these mechanoreceptors facilitates the precise detection and modulation of sensory systems, ultimately influencing environmental interaction.

Tomiate, et al.^46^ reports the identification of telocytes (TCs) in the oral mucosa of Guinea pigs, specifically within the palatine epithelium. Using light microscopy, transmission electron microscopy (TEM), and likely immunohistochemistry, the authors describe TCs with their characteristic ultrastructural features—small cell bodies, elongated telopodes with podoms and podomers, and heterochromatic nuclei. These cells form a three-dimensional network in the lamina propria, closely associated with blood vessels, nerves, and collagen fibers. The study highlights their preferential localization in mechanically stressed regions, such as the masticatory mucosa, suggesting a potential role in mechanosensation and tissue homeostasis. The findings align with previous reports of TCs in other tissues and species, reinforcing their widespread presence in vertebrates while expanding their known distribution to the oral cavity. The authors propose that TCs in this region may contribute to intercellular signaling, extracellular matrix remodeling, and tissue repair, opening new avenues for research into their functional roles in oral biology.

IHC used for identification of TCs and their roles in various tissues, including their interactions with surrounding structures such as nerves and mechanoreceptors. The use of specific markers like CD34, CD21, CD68, and VEGF in IHC provides valuable insights into the characteristics and functions of TCs.

CD34 is a well-established marker for identifying progenitor cells, including mesenchymal stem cells^47^ and vascular endothelial progenitor cells (pericytes)^48^. Expression of CD34 by stromal cells linked to tissue remodeling or repair^49^. CD34 serves as a marker to demonstrate the presence and localization of TCs^50^. In the current study, CD34-postive TCs identified around nerve fibers and sensory structures. The expression of CD34 in TCs is essential for understanding their role in tissue regeneration and wound healing processes^51,52^.

CD21 is a receptor for complement components and is primarily expressed on B lymphocytes^53^ and follicular dendritic cells^54^. Its expression on TCs can indicate their involvement in immune modulation and interactions with the immune system. Telocytes have been proposed to play roles in immune responses^9,55^and their expression of CD21 suggests a potential involvement in immunological processes within the nerve microenvironment.

Similarly, CD68 is a marker for macrophages and monocytes, and its expression can indicate the presence of inflammatory or immune cells^56^. Telocytes may interact with immune cells, facilitating the regulation of local inflammatory responses^51^. Using CD68 in IHC helps to identify the immunoreactivity in telocytes suggests potential immunomodulatory functions, as CD68 + cells have been reported to interact with T lymphocytes^57^. These reveal potential roles of TCs in immune surveillance, tissue repair, and regeneration.

Vascular endothelial growth factor (VEGF) is a crucial factor for angiogenesis and endothelial cell function, promoting the growth of new blood vessels^58^. In the context of TCs, VEGF expression can highlight their role in promoting vascularization around sensory and nerve structures. The association of TCs with VEGF underscores their potential contribution to tissue repair, particularly in areas that require new vascular growth, such as sites of nerve injury. The presence of VEGF in TCs suggests that these cells might play an active role in tissue regeneration by enhancing blood supply, which is critical for supporting nerve function and promoting wound healing.

The immunohistochemical profile of TCs reflects their function. CD34 highlights potential role of TCs for tissue regeneration and repair. CD21 and CD68 reveal the immune-modulatory roles of TCs, indicating their involvement in maintaining tissue homeostasis and responding to injury or inflammation. VEGF emphasizes the potential of TCs in promoting angiogenesis and supporting vascularization, which is vital for maintaining nerve function and tissue integrity during repair processes.

## Conclusion

The use of IHC with CD34, CD21, CD68, and VEGF in studying telocytes (TCs) provides a multifaceted understanding of their roles in the nerve microenvironment. This approach allows researchers to explore the structural, immune-modulatory, and vascular contributions of TCs, offering valuable insights into their therapeutic potential in conditions like nerve injury, inflammation, and tissue regeneration. By integrating these markers, researchers can assess the complex interactions between TCs, immune cells, blood vessels, and nerve structures, enabling the development of more informed strategies for targeted therapies in neurodegenerative diseases and traumatic nerve damage.

Telocytes (TCs) play a crucial role in supporting sensory nerve fibers and mechanoreceptors such as the Herbst and Ruffini corpuscles. Their close association with these structures helps maintain the integrity and function of sensory systems, ensuring responses to pressure, stretch, and vibration. The unique morphological features of TCs, including telopodes and podoms, allow them to interact with sensory nerve endings, enhancing their ability to maintain sensory responses. Based on the morphological evidence, TCs may contribute to sensory processing through: (1) structural support – maintaining the 3D organization of sensory nerve endings via their long telopodes; (2) mechanical signal modulation – potentially transmitting/distributing mechanical stimuli through their caveolae-rich processes (Popescu & Faussone-Pellegrini, 2010); and (3) neuron–glia-like interactions – suggested by close membrane contacts with sensory neurons, analogous to Schwann cell–axon relationships. These proposed functions align with demonstrated TC roles in other systems (Cretoiu et al., 2016), where they participate in intercellular signaling via extracellular vesicle release or gap junction communication. However, future functional studies are needed to confirm the exact mechanisms in sensory reception.

The clinical relevance of these findings is significant, as TCs are key players in nerve repair and regeneration. Their involvement in sensory modulation suggests potential therapeutic avenues for conditions related to nerve injury or sensory dysfunction. Ongoing research into the role of TCs will deepen our understanding of their functions in sensory systems and may pave the way for novel treatments for sensory disorders.

## Supplementary Information

Below is the link to the electronic supplementary material.

Supplementary Material 1

## Data Availability

Availability of data and material: The data sets collected and/or analyzed during the current study are available from the corresponding authors on reasonable request.
