# Supplementary figures and images for: Unveiling the role of perineural telocytes in mechanosensation, structural insights into their association with herbst and ruffini corpuscles in the quail beak
Source: Sci Rep. 2025 Sep 12;15:32431. doi: 10.1038/s41598-025-15900-1 (PMC12432264; doi:10.1038/s41598-025-15900-1)

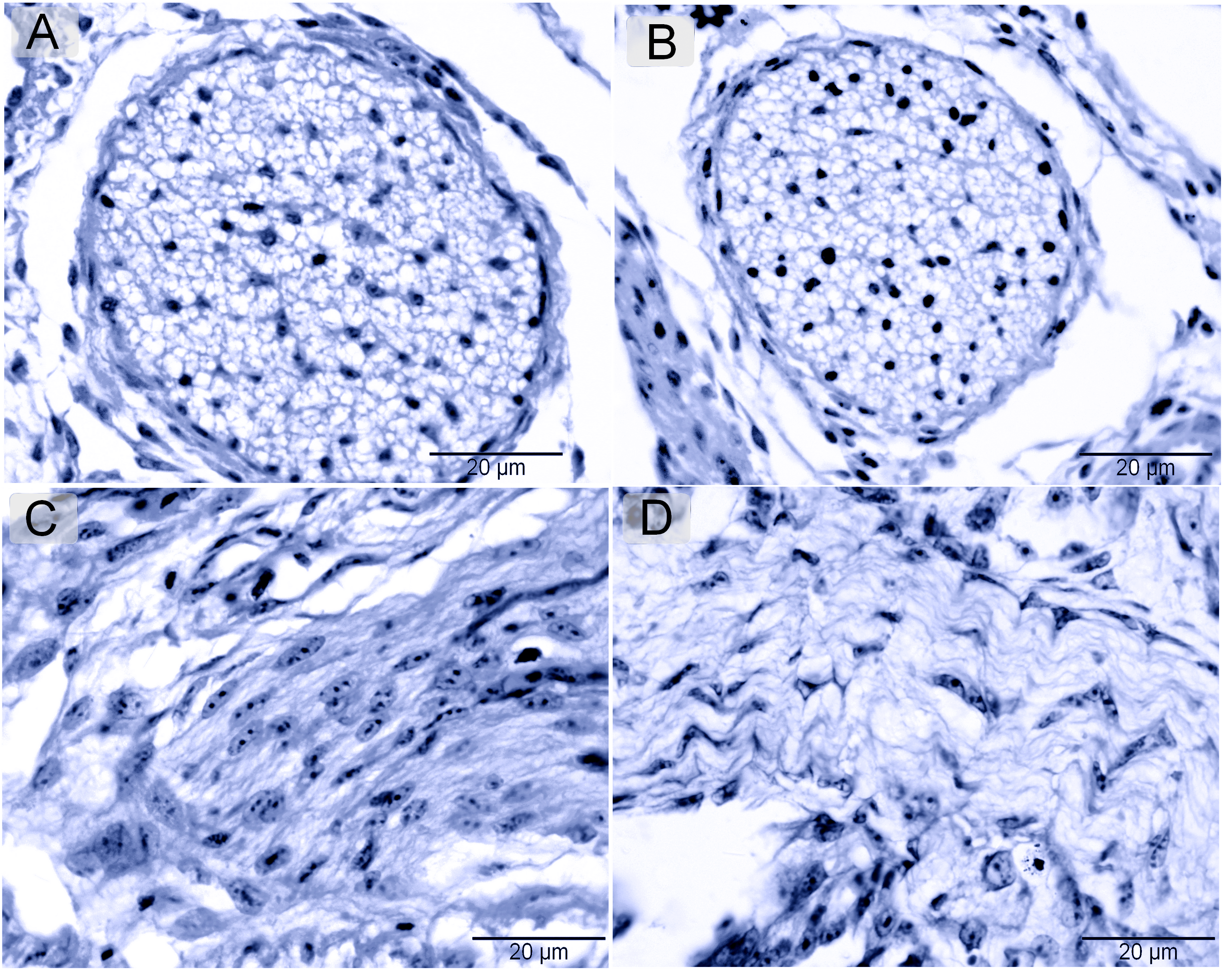


Negative control

Paraffin sections for negative control of CD34 (A), VEGF, (B), CD 21 (C), CD 68 (D)

Supplement: Supplementary file 1 — Supplementary Material 1 [file 41598_2025_15900_MOESM1_ESM.docx]
